# Inhibition of NAT10 Suppresses Melanogenesis and Melanoma Growth by Attenuating Microphthalmia-Associated Transcription Factor (MITF) Expression

**DOI:** 10.3390/ijms18091924

**Published:** 2017-09-07

**Authors:** Taek-In Oh, Yoon-Mi Lee, Beong-Ou Lim, Ji-Hong Lim

**Affiliations:** 1 Department of Biomedical Chemistry, College of Biomedical & Health Science, Konkuk University, Chungju 27478, Chungbuk, Korea; dk1050@kku.ac.kr (T.-I.O.); beongou@kku.ac.kr (B.-O.L.); 2 Department of Food Bioscience, College of Biomedical & Health Science, Konkuk University, Chungju 27478, Chungbuk, Korea; yoonmilee@kku.ac.kr; 3 Nanotechnology Research Center, Konkuk University, Chungju 27478, Chungbuk, Korea

**Keywords:** NAT10, remodelin, MITF, melanogenesis, melanoma growth

## Abstract

*N*-acetyltransferase 10 (NAT10) has been considered a target for the treatment of human diseases such as cancer and laminopathies; however, its functional role in the biology of melanocytes is questionable. Using a small molecule or small interfering RNA targeting NAT10, we examined the effect of NAT10 inhibition on melanogenesis and melanoma growth in human and mouse melanoma cells. Genetic silencing or chemical inhibition of NAT10 resulted in diminished melanin synthesis through the suppression of melanogenesis-stimulating genes such as those encoding dopachrome tautomerase (DCT) and tyrosinase in B16F10 melanoma cells. In addition, NAT10 inhibition significantly increased cell cycle arrest in S-phase, thereby suppressing the growth and proliferation of malignant melanoma cells in vitro and in vivo. These results demonstrate the potential role of NAT10 in melanogenesis and melanoma growth through the regulation of microphthalmia-associated transcription factor (MITF) expression and provide a promising strategy for the treatment of various skin diseases (melanoma) and pigmentation disorders (chloasma and freckles).

## 1. Introduction

*N*-acetyltransferase 10 (NAT10), a member of GCN5-related *N*-acetyltransferases (GNAT), has been reported to be involved in the regulation of ribosome biogenesis, histone acetylation, DNA damage response, microtubule reorganization, and cell cycle progression [1,2,3,4]. The expression of NAT10 is reported to increase in response to DNA damage, and ectopic expression of NAT10 maintains cell survival upon DNA damage stress [5]. Therefore, NAT10 may be involved in the biology of melanocyte, a cell subtype responsible for preventing skin disorder caused by ultraviolet (UV)-induced DNA damage. In addition, NAT10 is highly overexpressed in multiple tumor types, including melanoma, and overexpression of NAT10 correlates with worse outcomes in patients with hepatocellular carcinoma [6,7]. However, the functional roles of NAT10 in melanoma growth remain unknown. A recent study demonstrated that a small molecule, “remodelin”, targeting NAT10 restores nuclear shape of Hutchinson–Gilford progeria syndrome (HGPS)-derived patient cells via microtubule reorganization [1].

Microphthalmia-associated transcription factor (MITF) promotes melanogenesis in the presence of α-melanocyte-stimulating hormone (α-MSH). α-MSH is produced and secreted from UV-exposed keratinocytes through the transcriptional activation of several enzymes such as tyrosinase and dopachrome tautomerase (DCT), which are essential for melanin production and melanocyte differentiation [8,9]. In addition, MITF has been reported as an amplified oncogene in human melanomas and displays functional roles in transcriptional gene expression related to survival, proliferation, cell cycle progression, and chemoresistance [10]. MITF regulates the expression of numerous genes involved in the regulation of cell proliferation, such as B-cell lymphoma 2 (BCL2), cyclin-dependent kinase 2 (CDK2), cyclin D1, and p21, and promotes cell proliferation in melanocytes and melanomas [9,11,12]. As MITF is an oncogenic driver in melanomas, identification of therapeutic strategies targeting MITF or its target genes is clinically important for the treatment of melanoma. Previous reports have shown that several small molecules such as HDAC inhibitors, CH5552074, and ML329 targeting MITF reduced the expression of genes involved in pigmentation and cell cycle regulation in primary melanocytes and differentiated melanoma cells [13,14,15].

In the present study, we investigate the functional role of NAT10 in melanogenesis and melanoma growth. This is the first study to suggest that the pharmacological or genetic inhibition of NAT10 significantly suppresses α-MSH–stimulated melanin synthesis through the downregulation of MITF target genes related to pigmentation. Moreover, we show that the suppression of NAT10 reduces melanoma growth with abnormal expression of cell cycle-regulating genes such as CDK2 and cyclin D1 in vitro and in vivo.

## 2. Results

### 2.1. Remodelin Attenuates Melanin Synthesis by Repressing MITF (Microphthalmia-Associated Transcription Factor)

**Figure 1:**
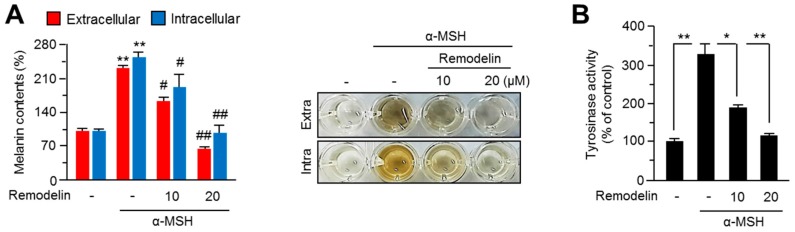

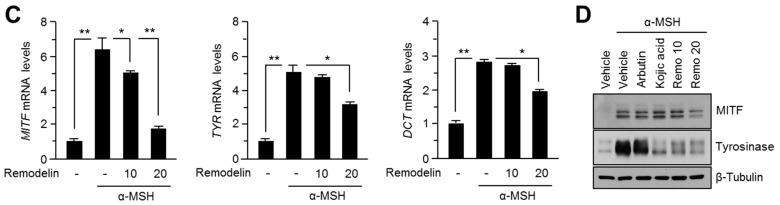
Effect of remodelin on melanin synthesis in B16F10 mouse melanoma cells. (**A**) Remodelin suppressed α-melanocyte-stimulating hormone (α-MSH)-induced melanin synthesis. Cells were pre-incubated in the absence or presence of remodelin for 1 h, followed by their incubation with α-MSH (0.2 mM) for 3 days. α-MSH increased the extracellular and intracellular melanin content; however, remodelin treatment decreased these levels. Color changes in the cultured medium are shown. Values represent mean ± SD of three independent experiments performed in triplicates; ** *p* < 0.01, # *p* < 0.05, and ## *p* < 0.01; (**B**) Inhibitory effect of remodelin on cellular tyrosinase activity. Tyrosinase activity was determined by measuring l-DOPA (2 mM) oxidation to dopachrome at 475 nm (OD475). Values represent mean ± SD of three independent experiments performed in duplicates; * *p* < 0.05 and ** *p* < 0.01; (**C**) Effect of remodelin on MITF, TYR, and DCT mRNA expression levels was measured using quantitative RT-PCR. Values represent mean ± SD of three independent experiments performed in duplicates; * *p* < 0.05 and ** *p* < 0.01; (**D**) Effect of remodelin on MITF and tyrosinase protein levels. B16F10 cells were pre-incubated with arbutin, kojic acid, or remodelin for 1 h, followed by their treatment with α-MSH for 12 h. MITF and tyrosinase protein expression levels were measured by western blotting.

To elucidate the effects of the pharmacological inhibition of NAT10 on melanin synthesis, we initially analyzed melanin contents in B16F10 mouse melanoma cells-treated with remodelin at different concentrations. α-melanocyte-stimulating hormone (α-MSH)-mediated increase in the extracellular and intracellular melanin contents was significantly decreased upon treatment with 20 µM remodelin (Figure 1A). As tyrosinase is a key enzyme involved in melanin production through l-3,4-dihydroxyphenylalanine (l-DOPA) oxidation in melanocytes, we further measured the cellular tyrosinase activity. Tyrosinase activity was largely decreased by remodelin (Figure 1B). To elucidate the mechanism underlying remodelin-mediated decrease in melanin synthesis, we investigated the effect of remodelin on the expression of MITF and its pigmentation-related target genes, which play key roles in melanin synthesis. As shown in Figure 1C, the expression of pigmentation-related genes such as MITF, tyrosinase, and DCT was decreased following remodelin treatment. In addition, protein levels of MITF and tyrosinase were decreased in remodelin-treated B16F10 cells (Figure 1D). These results suggest that the pharmacological inhibition of NAT10 suppresses melanogenesis through the downregulation of MITF and its melanogenic target genes.

### 2.2. Genetic Silencing of NAT10 Suppresses Melanin Synthesis

**Figure 2:**
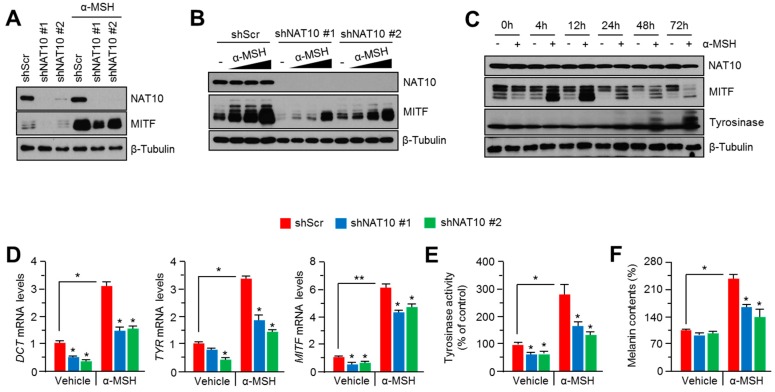
Inhibitory effect of genetic silencing of NAT10 on melanin synthesis in B16F10 cells. (**A**) Effect of NAT10 knockdown on MITF and NAT10 protein expression levels. Cells were treated with α-MSH for 8 h prior to the experiment. Indicated protein levels were measured by western blotting; (**B**) NAT10 knockdown attenuated MITF accumulation in response to α-MSH. B16F10 cells were infected with short hairpin RNA against NAT10 or control and stable cells were incubated with α-MSH in a dose-dependent manner (0, 50, 100, and 200 nM); (**C**) α-MSH treatment showed no effect on NAT10 expression in B16F10 cells. B16F10 cells were treated with α-MSH in a time-dependent manner and NAT10, MITF, and tyrosinase proteins were measured by western blotting; (**D**) Inhibitory effect of NAT10 knockdown on DCT, TYR, and MITF mRNA expression. Cells were incubated with α-MSH for 12 h and the indicated mRNA levels measured by quantitative RT-PCR. Values represent mean ± SD of three independent experiments performed in duplicates; * *p* < 0.05 and ** *p* < 0.01; (**E**) Inhibitory effect of NAT10 knockdown on cellular tyrosinase activity. Cells were incubated with α-MSH for 3 days prior to tyrosinase activity assay. Values represent mean ± SD of three independent experiments performed in triplicates; * *p* < 0.05; (**F**) Inhibitory effect of NAT10 knockdown on melanin synthesis. Cells were incubated with α-MSH for 3 days prior to the measurement of melanin contents. Values represent mean ± SD of three independent experiments performed in duplicates; * *p* < 0.05.

To investigate whether NAT10 is required for melanin synthesis, we generated B16F10 cells with stable knockdown of NAT10 expression. As shown in Figure 2A, NAT10 suppression resulted in the downregulation of MITF. In addition, MITF expression induced by α-MSH was significantly reduced in a dose-dependent manner in NAT10-silenced B16F10 cells (Figure 2B). Next, we evaluated whether α-MSH affects NAT10 expression in the early stage of MITF-mediated melanogenic signaling pathway. Figure 2C shows that α-MSH stimulation had no effect on NAT10 expression but increased the expression of MITF and tyrosinase (as positive controls), suggesting that α-MSH stimulation has no direct effect on NAT10 expression. As MITF is downregulated in NAT10-silenced cells, melanogenesis-related MITF target genes were measured in the absence or presence of α-MSH. As observed in Figure 2D, the expression of MITF target genes such as DCT and tyrosinase was significantly decreased in NAT10-knockdown cells. Suppression of cellular tyrosinase activity and intracellular melanin contents was observed upon α-MSH stimulation in NAT10-silenced B16F10 cells (Figure 2E,F). These results reveal that NAT10 may partly control melanin synthesis by regulating MITF expression.

### 2.3. Inhibition of NAT10 Delays Cell Cycle Progression via S-Phase Arrest

**Figure 3:**
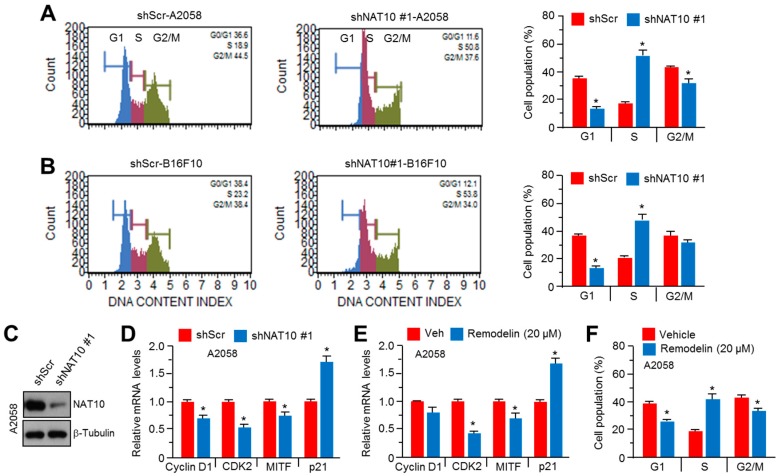
NAT10 knockdown causes S-phase cell cycle arrest. (**A**,**B**) A2058 and B16F10 cells were infected with lentivirus carrying short hairpin RNA against NAT10 or control and the infected cells were selected by puromycin for 4 days. After puromycin selection, cells were cultured and cell cycle proportions were determined using Muse™ Cell Cycle Kit. Values represent mean ± SD of two independent experiments performed in triplicates; * *p* < 0.05; (**C**) NAT10 knockdown efficiency in A2058 human melanoma cells; (**D**) Effect of NAT10 knockdown on the expression of genes related to cell cycle progression. Indicated gene expression levels were measured by quantitative RT-PCR. Values represent mean ± SD of three independent experiments performed in duplicates; * *p* < 0.05; (**E**) Effect of remodelin on the expression of genes related to cell cycle progression. Cells were incubated in the absence or presence of remodelin for 48 h and gene expression levels were measured by quantitative RT-PCR. Values represent mean ± SD of three independent experiments performed in duplicates; * *p* < 0.05; (**F**) Effect of remodelin on cell cycle progression. Cells were incubated in the absence or presence of remodelin for 3 days and cell populations were analyzed by Muse™ Cell Cycle Kit. Values represent mean ± SD of three independent experiments performed in duplicates; * *p* < 0.05.

It is known that MITF is closely linked to cell cycle progression in malignant melanoma growth [9]. However, the effect of the pharmacological or genetic inhibition of NAT10 on cell cycle progression in human or mouse melanoma cells is questionable. To investigate this, we knocked down NAT10 expression in A2058 human malignant melanoma cells (Figure 3C). As shown in Figure 3A,B, the population of cells arrested at S-phase was significantly increased in NAT10-silenced A2058 and B16F10 cells. In addition, the number of cells arrested at S-phase was high in remodelin-treated A2058 cells (Figure 3F). To elucidate how NAT10 suppression delays cell cycle progression, we further analyzed the expression of genes related to cell cycle progression (MITF target genes). Cyclin D1 and CDK2, which promote cell cycle progression at G1 or S phase, were significantly downregulated along with MITF in NAT10-silenced or remodelin-treated A2058 cells (Figure 3D,E). In addition, the pharmacological or genetic inhibition of NAT10 increased the expression of p21, an endogenous inhibitor of G1/S transition (Figure 3D,E). These results suggest that NAT10 may be associated with malignant melanoma growth through MITF/cyclin D1/CDK2/p21-mediated cell cycle progression.

### 2.4. Inhibition of NAT10 Delays Melanoma Growth In Vitro and In Vivo

**Figure 4:**
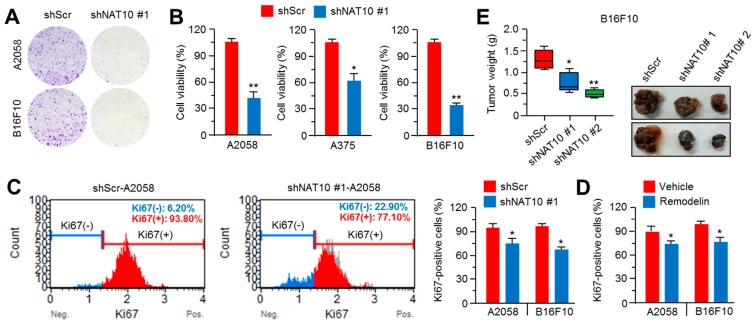
NAT10 knockdown attenuates melanoma cell growth in vitro and in vivo. (**A**) Suppressive effect of NAT10 knockdown on melanoma cell growth. Control- or NAT10 knockdown A2058 or B16F10 cells were incubated for 7 days, followed by their staining with crystal violet; (**B**) Control- or NAT10-knockdown cells were cultured for 3 days and their viability measured by crystal violet assay. Values represent mean ± SD of three independent experiments performed in duplicates; * *p* < 0.05 and ** *p* < 0.01; (**C**) Suppressive effect of NAT10 knockdown on melanoma cell proliferation. A2058 and B16F10 cells were infected with lentivirus carrying short hairpin RNA against NAT10 or control and the infected cells were selected by puromycin for 4 days. After puromycin selection, cells were cultured and Ki-67–positive cells were determined using Muse™ Ki-67 Assay Kit. Values represent mean ± SD of two independent experiments performed in triplicates; * *p* < 0.05 (right panel); (**D**) Effect of remodelin on melanoma cell proliferation. A2058 and B16F10 cells were incubated in the absence or presence of remodelin for 3 days and Ki-67–positive cells determined using Muse™ Ki-67 Assay Kit. Values represent mean ± SD of two independent experiments performed in triplicates; * *p* < 0.05; (**E**) Three weeks after the injection of NAT10-knockdown B16F10 cells, mice were sacrificed and tumor weights measured. Final tumor weights were represented in the box plots (*n* = 5). The whiskers in the box plots represent the maximum and the minimum value; * *p* < 0.05 and ** *p* < 0.01. Tumor images were shown.

Next, we evaluated the growth of NAT10-silenced A2058 and B16F10 cells. The clonogenic cell growth was dramatically decreased in NAT10-silenced cells (Figure 4A). In addition, reduced viability was observed in NAT10-silenced A2058, A375, and B16F10 cells (Figure 4B). As retardation of cell proliferation is caused by S-phase arrest, we analyzed Ki-67-positive cell population. As shown in Figure 4C,D, the population of proliferating Ki-67-positive cells was decreased by approximately 10–20% in NAT10-silenced and remodelin-treated cells. We examined the anti-tumor growth effect of NAT10 inhibition in vivo to determine if NAT10 may promote melanoma growth through the regulation of cell cycle progression. We found that the growth of xenografted tumor derived from NAT10-silenced B16F10 cells was strongly reduced (Figure 4E). These results indicate that NAT10 may play an important role as a tumor-promoting factor in malignant melanoma.

## 3. Discussion

The pharmacological inhibition of NAT10 rescues nuclear architecture via microtubule reorganization in laminopathic cells [1]. Several reports have shown that the overexpression of NAT10 promotes cancer growth and metastasis and results in worse outcomes in patients with the liver and colorectal cancers [3,7,16]. Here, we found that NAT10 knockdown induces cell cycle arrest and suppresses cell proliferation along with the expression of MITF and its target genes related to cell cycle progression in mouse and human melanoma cells. Consistent with our findings, clinical evidence has reported that NAT10 is partly overexpressed in melanoma tissues as well as neuroblastoma and prostate carcinoma derived from patients [6]. Therefore, these results suggest that NAT10 may have functional role in oncogenesis at least in some cancer types.

Environmental stresses such as oxidative stress and DNA damage by genotoxic agents are known to increase NAT10 gene expression via transcriptional activation of its promoter, suggesting that overexpression of NAT10 may confer protective effect to DNA damage or oxidative stress [5]. Whether NAT10 acts as a critical factor in the biology of melanocytes is still unclear. Our results, show, for the first time, that inhibition of NAT10 decreased melanin synthesis via downregulation of MITF and its melanogenic target genes. These observations suggest the pigmentation-related role of NAT10 in melanocytes as a response to α-melanocyte-stimulating hormone (α-MSH) induced by UV exposure. 

We observed that NAT10 knockdown decreased cell survival and proliferation in vitro and in vivo, which is contradictory to a previous report, wherein NAT10 suppression increased cell survival and prevented DNA damage-induced apoptosis in U2OS and HCT116 cells [2]. Therefore, the functional role of NAT10 in determination of cell fate may be dependent on MITF expression. 

Our results show that the pharmacological or genetic inhibition of NAT10 causes downregulation of MITF expression. However, it would be interesting to investigate the precise molecular mechanism by which NAT10 regulates MITF expression. We propose two plausible hypothesis. First, NAT10 may act as a transcriptional activator of MITF promoter alone or in cooperation with several transcription factors, including cAMP response element-binding protein (CREB), paired box 3 (PAX3), SRY-related HMG-box 10 (SOX10), and lymphoid enhancing factor-1 (LEF-1), which are known as upstream transcription regulators of MITF [17]. Alternatively, NAT10 may regulate post-translational modification such as *N*-terminal acetylation of MITF or its upstream transcription regulators. NAT10 belongs to GNAT family of histone acetyltransferases (HATs), which bear a HAT domain. A recent report revealed that NAT10 directly acetylates non-histone proteins such as p53 and upstream binding factor (UBF) [2,18]. Moreover, post-translational modifications of MITF such as sumoylation and phosphorylation that alter its transcriptional activity have been reported [19,20]. These reports support the possible hypothesis that MITF directly affects its transcriptional activity or stability via acetylation by NAT10. 

Taken together, the presented data reveal that the inhibition of NAT10 by small molecules or genetic silencing suppresses melanin synthesis and melanoma growth by decreasing the expression of MITF and its target genes related to pigmentation and cell proliferation. Our results provide new clues to elucidate the biological function of NAT10 in differentiation and proliferation of melanocyte and melanoma and helpful information for the development of therapeutic strategy for treating skin diseases such as melanoma and hyperpigmentation disorders. 

## 4. Materials and Methods

### 4.1. Reagents and Antibodies

Remodelin (S7641), α-MSH (M4135), arbutin (A4256), kojic acid (K3125), and l-DOPA (333786) were purchased from Selleckchem (Houston, TX, USA) and Sigma-Aldrich (St. Louis, MO, USA). Antibody against MITF (#12590) was obtained from Cell Signaling Technology (Danvers, MA, USA). Antibodies against tyrosinase (sc-7833) and β-tubulin (sc-9104) were supplied by Santa Cruz Biotechnology (Dallas, TX, USA).

### 4.2. Cell Culture and Generation of Stable Cell Lines

Dulbecco’s modified Eagle’s medium (DMEM) containing 10% fetal bovine serum (FBS) and 1% penicillin-streptomycin was used to culture B16F10 and A2058 melanoma cell lines. The pLKO.1-shNAT10 silencing mouse NAT10 (TRCN0000184712 and TRCN00001842285) and human NAT10 (TRCN0000296354) were purchased from Sigma-Aldrich and used to knockdown mouse and human NAT10 in B16F10 and A2058 cells, respectively. Lentiviral particles encoding short hairpin RNA were generated in HEK293T cells. Briefly, pLKO.1-shRNA and packaging vectors, pMD2G and psPAX2, were transfected into HEK293T cells and incubated for 3 days. Lentivirus containing cultured medium was introduced into B16F10 and A2058 melanoma cells, and then the infected cells were further incubated and selected using 2 µg/mL puromycin (Invitrogen, Carlsbad, CA, USA).

### 4.3. Western Blotting

Protein samples were prepared using lysis buffer contained 1% IGEPAL, 150 mM sodium chloride (NaCl), 50 mM Tris-HCl (pH 7.9), 10 mM sodium fluoride (NaF), 0.1 mM ethylenediaminetetraacetic acid (EDTA), and protease inhibitor cocktail. Total protein samples (30 µg) were subjected to sodium dodecyl sulfate polyacrylamide gel electrophoresis (SDS-PAGE). Polyvinylidene fluoride (PVDF) membrane (Millipore, Billerica, MA, USA) was used to western blotting. Primary antibodies (1:1000–5000) diluted in 5% BSA were reacted for overnight at 4 °C, and then horseradish peroxidase-conjugated secondary antibodies (1:10,000) for 1 h at room temperature. Enhanced chemiluminescence (ECL) prime kit (GE healthcare, Pittsburgh, PA, USA) was used to visualize the protein expression.

### 4.4. Determination of Melanin Contents

Melanin contents were analyzed as previously described [21,22]. Mouse melanoma cell line, B16F10 cells were cultured into 6 well culture plate (1 × 10^5^ cells/well) and incubated with α-MSH in the absence or presence of remodelin (10 and 20 µM) for 3 days. The harvested cells or media were centrifuged and the pellets were solubilized in 1 N sodium hydroxide (NaOH) at 80 °C for 1 h. The melanin contents were measured using spectrophotometer at 475 nm wavelength (OD475) (BioTek, Winooski, VT, USA) and the values normalized to the cellular protein concentration.

### 4.5. RNA Isolation and Quantitative Reverse Transcription Polymerase Chain Reaction (RT-PCR)

RNA isolation was performed as previously described [23]. Total RNA was isolated from cultured cells by TRIzol (Invitrogen) and 2 µg of fresh RNA was used for cDNA synthesis by a high capacity cDNA reverse transcription kit (Applied Biosystems, Foster city, CA, USA). Quantitative real-time PCR was performed using SYBR Green PCR Master Mix (Applied Biosystems). The sequences of the PCR primers (5′–3′) were as follows: TCAAGTTTCCAGAGACGGGT and CATCATCAGCCTGGAATCAA for MITF; ATAGGTGCATTGGCTTCTGG and TCTTCACCATGCTTTTGTGG for tyrosine; CTCATCAAAGATGGCGTCTG and CTTCCTGAATGGGACCAATG for DCT; GGCGGATTGGAAATGAACTT and TCCTCTCCAAAATGCCAGAG for cyclin D1; GAATCTCCAGGGAATAGGGC and CTGAAATCCTCCTGGGCTG for CDK2; GAGAAATCAAACAGAGGCCG and CTGAGTACCTGAACCGGCA for BCL2; CATGGGTTCTGACGGACAT and AGTCAGTTCCTTGTGGAGCC for p21.

### 4.6. Tumor Xenograft Assay

Tumor xenograft assay was performed as previously described [23]. B16F10 mouse melanoma cells (1 × 10^6^) were subcutaneously injected into the flank of Balb/c-nude mice in 100 µL of FBS-free media. Final tumor weights were measured and calculated after 3 weeks of cell injection. The animal experiments were conducted and managed in accordance with the guidelines of the Konkuk University Institutional Animal Care and Use Committee (21-09-2015, KU15072-1).

### 4.7. Cellular Tyrosinase Activity Assay

Cellular tyrosinase activity assay was performed as previously described [21]. Briefly, B16F10 cells were cultured into a six-well culture plate (1 × 10^5^ cells/well) and incubated in the absence or presence of remodelin and α-MSH. After incubation, the cells were washed and lysed using cold phosphate-buffered saline (PBS) and 1% Triton X-100. Protein concentration was measured using Bradford assay (Bio-Rad, Hercules, CA, USA, #500-0006). 50 µg of total protein samples were mixed with 2 mM l-DOPA, and then oxidized l-DOPA was measured every 5 min for 1 h when color change was observed at 475 nm (OD475). Values after 15 min reaction were obtained and normalized to protein concentration. 

### 4.8. Measurement of Cell Viability and Clonogenic Assay

To measure cell viability, cells were seeded and incubated into 24- (1 × 10^5^ cells) or 96- (2 × 10^4^ cells) well tissue culture plates. Cells were washed and stained in cold PBS and 0.5% crystal violet staining solution. For clonogenic assay, A2058 and B16F10 cells were seeded into six-well tissue culture plates at concentration estimated to yield 50 colonies/well and incubated for 6 days. After incubation, cultured cells were washed with PBS, fixed with 4% paraformaldehyde, and incubated in 0.5% crystal violet staining solution for 20 min at room temperature. The stained cells were lysed using 1% SDS solution, and then optical density was measured at 570 nm (OD570) using an absorbance reader (BioTek).

### 4.9. Cell Cycle and Proliferation Assay

A2058 and B16F10 stable cells were seeded at a density 1 × 10^5^ cells/well into a six-well cell culture plate. Cell cycle analysis was performed according to the supplier's instructions (Millipore, Cat No. MCH100106). The cells were washed with PBS and centrifuged at 2000 rpm for 2 min at room temperature. The pellets were fixed with 70% ice-cold ethanol and incubated at −20 °C for 3 h. After fixation, cells were incubated with 200 µL Muse™ cell cycle assay kit reagent for 30 min at room temperature, and then cells were subjected to a Mini Flow Cytometry Muse™ Cell Analyzer (EMD Millipore). For Ki-67 cell proliferation assay, NAT10-knockdown A2058 and B16F10 cells (1 × 10^5^ cells/well) were cultured into a six-well tissue culture plate. The cultured cells were collected and fixed using fixative solution for 15 min, and then incubated with 100 µL permeabilization solution for 15 min at room temperature. After permeabilization, 50 µL of the sample and assay buffer were mixed and treated with 10 µL Muse™ Hu Ki-67 antibody for 30 min at room temperature in the dark. After incubation, 150 µL of 1× assay buffer was added to each sample and the samples analyzed using a Mini Flow Cytometry Muse™ Cell Analyzer (EMD Millipore).

### 4.10. Statistical Analysis

GraphPad Prism (GraphPad Software Inc., La Jolla, CA, USA) used to perform statistical analysis. The unpaired Student’s *t*-test was performed for two experimental comparisons and one-way ANOVA with Tukey post-test was performed for multiple comparisons. Data are represented as mean ± standard deviation (SD). Statistical significance was considered when *p* < 0.05. 

## 5. Conclusions

In the present study, we provide several major findings as follows: genetic silencing or chemical inhibition of NAT10 (i) inhibits melanogenesis stimulated by α-MSH through downregulation of MITF and its pigmentation-related genes; (ii) suppresses melanoma growth and survival with decreased MITF and its target genes expression related to cell cycle regulation. These results suggest that NAT10 has a functional role in the physiological processes of melanocyte and melanoma and might be a therapeutic target for human skin diseases such as melanoma and pigmentation-related disorders. 

## Abbreviations

α-MSH	Alpha-melanocyte stimulating hormone
NAT10	*N*-acetyltransferase 10
MITF	Microphthalmia-associated transcription factor
GNAT	GCN5-related *N*-acetyltransferases
BCL2	B-cell lymphoma 2
DCT	Dopachrome tautomerase
UV	Ultraviolet
HGPS	Hutchinson-Gilford progeria syndrome
l-DOPA	l-3,4-dihydroxyphenylalanine
CREB	cAMP response element-binding protein
PAX3	Paired box 3
SOX10	SRY-related HMG-box 10
LEF-1	Lymphoid enhancing factor-1
UBF	Upstream binding factor
HAT	Histone acetyltransferase
CDK2	Cyclin-dependent kinase 2
